# Ecological determinants of stability in dryland plant communities

**DOI:** 10.1111/nph.71453

**Published:** 2026-07-29

**Authors:** Johanne Gresse, Jules Segrestin, Lars Götzenberger, Jan Lepš, Pierre Liancourt, Merav Seifan, Katja Tielbörger, Maria Májeková

**Affiliations:** ^1^ Plant Ecology Group, Institute of Evolution and Ecology University of Tübingen Tübingen 72076 Germany; ^2^ Department of Botany, Faculty of Sciences University of South Bohemia České Budějovice 370 05 Czech Republic; ^3^ Institute of Botany of the Czech Academy of Sciences Třeboň 379 01 Czech Republic; ^4^ Institute of Entomology Czech Academy of Sciences České Budějovice 370 05 Czech Republic; ^5^ Botany Department State Museum of Natural History Stuttgart Stuttgart 70191 Germany; ^6^ Mitrani Department of Desert Ecology, Swiss Institute for Dryland Environmental and Energy Research, The Jacob Blaustein Institutes for Desert Research Ben‐Gurion University of the Negev Midreshet Ben‐Gurion 8499000 Israel; ^7^ Cluster of Excellence (EXC 3121): TERRA – Terrestrial Geo‐Biosphere Interactions in a Changing World University of Tübingen Tübingen 72076 Germany; ^8^ Cluster of Excellence GreenRobust Eberhard Karls University Tübingen 72076 Germany

**Keywords:** aridity, asynchrony, biodiversity, community dynamics, drylands, temporal stability, unpredictability

## Abstract

- Community stability is often presented as crucial for sustaining ecosystem services under environmental change. Yet, its determinants (dominance, asynchrony in species fluctuations, averaging effect of species diversity) have rarely been disentangled in natural systems, and their contributions across climates remain unclear.
- We disentangled the determinants of stability in 40 winter annual communities comprising 178 populations across four climates (arid, semi‐arid, Mediterranean, mesic‐Mediterranean) and two microhabitats (open areas and under perennial shrubs) over 13 yr in a dryland biodiversity hotspot.
- Asynchrony in species fluctuations and the averaging effect of species diversity emerged as the strongest stabilizing mechanisms. Asynchrony consistently stabilized communities across climates, independent of species richness, challenging the ‘Insurance Hypothesis’. The averaging effect was weaker in desert and mesic‐Mediterranean climates, suggesting biodiversity may not always determine stability.
- These findings stress the need to separate compensatory dynamics into asynchrony in species fluctuations and the averaging effect of species diversity to better predict community stability over time under increasing climatic variability.

## Graphical abstract

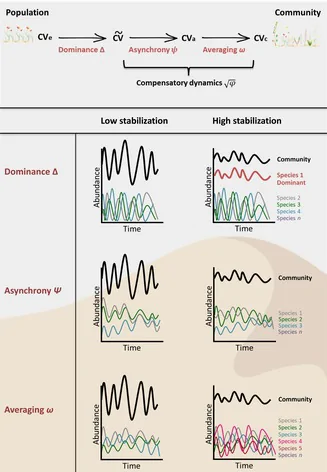

## Introduction

Drylands support a wealth of uniquely adapted species and play a crucial role in global biodiversity (Reynolds *et al*., 2007; Maestre *et al*., 2021; Wang *et al*., 2023; Gross *et al*., 2024). Their ability to provide key ecosystem services is increasingly threatened by climate change (Drori *et al*., 2021; Wang *et al*., 2024) and largely relies on the temporal stability of their communities (Bai *et al*., 2004; de Bello *et al*., 2021; Abel *et al*., 2024). Temporal stability is the degree to which a property of the community remains constant over time, measured as the inverse of temporal variability (Doak *et al*., 1998; de Bello *et al*., 2021; Segrestin & Lepš, 2022). Community stability primarily emerges from the stability of individual species' populations (de Bello *et al*., 2021; Lisner *et al*., 2024; Segrestin *et al*., 2024) and has been described as the outcome of three mechanisms operating among species: (1) the dominance effect, where more stable dominants drive overall community stability (Taylor, 1961; Grime, 1998; Sasaki & Lauenroth, 2011; de Bello *et al*., 2021; Hou *et al*., 2023; Lisner *et al*., 2023; Segrestin *et al*., 2024; Gracia *et al*., 2026), (2) the asynchrony in species fluctuations, leading to temporal compensations in biomass among species (Gonzalez & Loreau, 2009; Hector *et al*., 2010; Loreau & de Mazancourt, 2013; Blüthgen *et al*., 2016; Craven *et al*., 2018; Lepš *et al*., 2018; Valencia *et al*., 2020; de Bello *et al*., 2021; Lisner *et al*., 2024; Segrestin *et al*., 2024), and (3) the averaging effect of species diversity stabilizes total biomass more at the same level of asynchrony (Doak *et al*., 1998; Isbell *et al*., 2009; de Bello *et al*., 2021; Lisner *et al*., 2024; Segrestin *et al*., 2024).

Methodological limitations have prevented a clear disentanglement of their respective contributions to stability (Segrestin *et al*., 2024). Most studies combined asynchrony in species fluctuations and the averaging effect of species diversity under the broad term ‘compensatory dynamics’ and hypothesized them as the primary drivers of community stability (Isbell *et al*., 2009; Hector *et al*., 2010; Hautier *et al*., 2014; Blüthgen *et al*., 2016; Craven *et al*., 2018; Valencia *et al*., 2020). A prevalent assumption has been that negative correlations among species (antisynchrony) are more common in species‐rich communities (Isbell *et al*., 2009; Hou *et al*., 2023) as the chance of having species that respond differently to the environment increases with the number of species (the ‘Insurance hypothesis’; Loreau *et al*., 2021). However, this assumption is currently being debated (Valencia *et al*., 2020; Lisner *et al*., 2024). In contrast to compensatory dynamics, the alternative hypothesis that dominant species primarily drive community stability independently of species richness (dominance effect; Grime, 1998) has received comparatively less attention (Sasaki & Lauenroth, 2011; Ren *et al*., 2023; Lisner *et al*., 2024; Xu *et al*., 2026). A comprehensive analysis requires examining all three mechanisms concurrently.

Investigating long‐term community dynamics in natural environments is essential, as multiple abiotic factors, such as mean rainfall, its variability and unpredictability likely shape population stability and the combination of dominance, asynchrony and averaging effects (Bai *et al*., 2004; Craven *et al*., 2018; Ren *et al*., 2023). Moderate climate variability is thought to promote species asynchrony and richness by enabling temporal niche partitioning and species with distinct environmental responses (Gilbert *et al*., 2020), whereas extreme climate variability is predicted to synchronize species responses via shared stress and local extinctions, ultimately reducing diversity and destabilizing communities (Chesson & Huntly, 1989; Adler & Drake, 2008; Abrams *et al*., 2013). Arid populations and communities often show lower stability than in mesic climates due to scarce, highly variable and unpredictable precipitation (Noy‐Meir, 1973; Tielbörger *et al*., 2012, 2014; Wang *et al*., 2024; Gresse *et al*., 2026a).

Moreover, microhabitats within a climate can alter or buffer local conditions (Gilbert *et al*., 2020; Gresse *et al*., 2026a). In deserts, perennial shrubs tend to enhance nutrient availability and buffer rainfall variation and temperatures (Tielbörger & Kadmon, 2000; Liancourt *et al*., 2012; Castellanos *et al*., 2014; Tielbörger *et al*., 2014), thus increasing productivity, species richness and community stability compared with adjacent open areas (Holzapfel *et al*., 2006; Butterfield, 2009). As described by the stress‐gradient hypothesis, these positive shrub effects are expected to be strongest under harsh conditions, where facilitation prevails, and to weaken or shift toward competition as environmental stress decreases (Bertness & Callaway, 1994). Accordingly, in more mesic climates, microhabitats under shrubs may not improve environmental conditions and might even limit richness and productivity of understory communities (Holzapfel *et al*., 2006), for instance by reducing soil moisture (Tielbörger *et al*., 2014) and intensifying resource competition (Mndela *et al*., 2023). However, limited *in situ* data have constrained our understanding of the specificity or generality of the mechanisms underlying community stability (Gilbert *et al*., 2020; Ren *et al*., 2023). An ideal study system would span a natural gradient of habitats differing in their mean environmental conditions, as well as in their variability and unpredictability.

We disentangled the contribution of the three ecological mechanisms (dominance, asynchrony in species fluctuations and averaging effect of species diversity) to the stability of *in situ* annual dryland plant communities over 13 yr across four climates differing in rainfall annual mean, inter‐annual variability and unpredictability (arid – A, semi‐arid – SA, Mediterranean – M and mesic‐Mediterranean – MM), and across two distinct microhabitats (underneath and away from perennial shrubs in each climate). We used the year‐to‐year coefficient of variation (CV) of community biomass to assess stability and partitioned it into its four underlying mechanisms.

## Materials and Methods

### Environmental conditions

The research was conducted in Israel, within the Eastern Mediterranean region. The region is characterized by a winter growing season, and a prolonged summer drought. Four sites (mesic‐Mediterranean – MM, Mediterranean – M, semi‐arid – SA and arid – A) were selected along a steep gradient of aridity, rainfall variability and unpredictability from Northern to Southern Israel (Fig. 1). Aridity was quantified using mean annual precipitation (MAP) and the aridity index (AI; Zomer *et al*., 2022). MAP decreases from 740 mm (MM) to 110 mm (A) (Fig. 1a,b), and AI decreases from 0.54 in MM to 0.07 in A (Fig. 1a). Inter‐annual variation in precipitation (CV × 100%) increases from 22% in MM to 51% in A (Tielbörger *et al*., 2014; Fig. 1a,b). Colwell's indices of predictability (P), constancy (C) and contingency (M) (Colwell, 1974; Tonkin *et al*., 2017) also vary along the gradient. Predictability (P), which reflects the relative certainty of knowing a rainfall state (e.g. drought) at a given time, increases from 0.27 in A to 0.34 in M and slightly decreases to 0.32 in MM (Fig. 1a). Constancy (C), measuring how consistently a rainfall state (e.g. drought) persists across vegetation seasons, decreases from 0.14 in A to 0.08 in M and MM (Fig. 1a). Contingency (M), which indicates how rainfall states align with specific periods within a season, increases from 0.13 in A to 0.26 in M, then slightly decreases to 0.25 in MM (Fig. 1a). Most of the other environmental conditions were similar among the sites, south‐facing slopes, calcareous bedrock and similar elevations (364–537 m asl) and temperatures (19.1–20.9°C), ensuring little confounding of precipitation with other abiotic environmental factors.

**Fig. 1:**
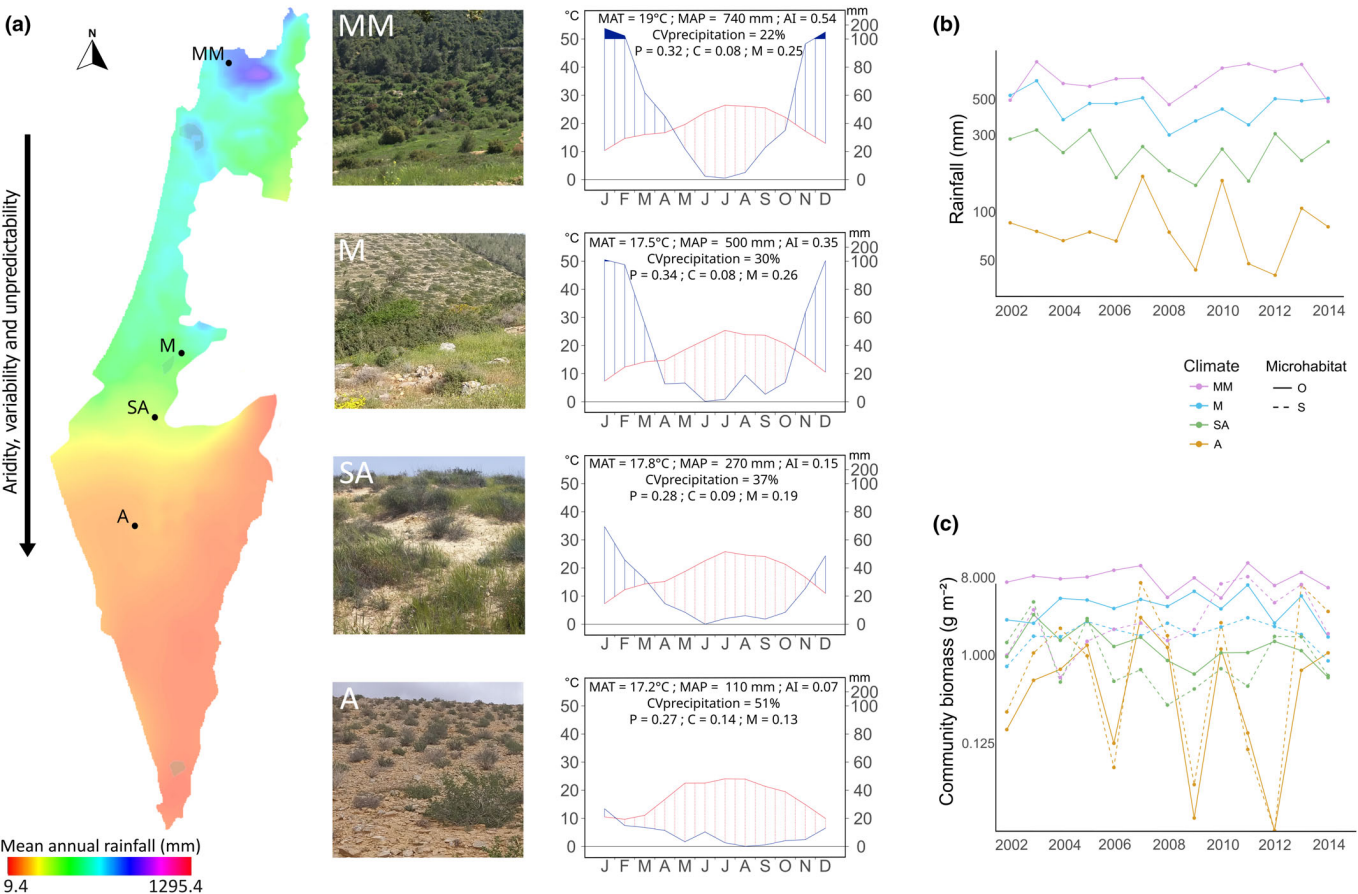
Overview of the study design and environmental context along the aridity gradient. (a) Location of the four field sites (MM, M, SA, A) along the mean annual precipitation gradient represented by the color gradient. The map layer has been extracted from BioGIS. Climate diagrams (cf. Walter *et al*., 1975) exhibiting monthly mean temperature (left *y*‐axis, red colored) and precipitation (right *y*‐axis, blue colored) for the 2002–2015 period. The axes are constructed so dotted areas identify drought periods (rainfall (mm) < 2 × temperature (°C)). Additionally, the mean annual temperature (MAT), mean annual precipitation (MAP), aridity index (AI), coefficient of variation in precipitation (%) and the Colwell's indices of predictability (predictability P, constancy C, contingency M) are reported in each panel. Rainfall, air temperature and evapotranspiration data were sourced from the Israeli Meteorological Service (IMS) and Ben Gurion University. (b) Scatterplot of the rainfall fluctuations (mm) in each site across the 13 yr (2002/2003–2014/2015). The scale was log transformed to better visualize the larger variation in annual rainfall toward the drier sites. (c) Scatterplot of the community biomass (Standing Biomass; g m^−2^) fluctuations in each habitat (i.e. in each site and microhabitat) across the 13 yr (2002/2003–2014/2015). The scale was log transformed to better visualize the larger variation in standing biomass toward the drier sites. A, Arid; M, Mediterranean; MM, mesic‐Mediterranean; SA, semi‐arid.

All sites are dominated by winter annuals, which are the focus of this study (Supporting Information Table S1). They make up 70–95% of plant diversity and up to 100% of ecosystem productivity – with increasing importance toward more arid climates (Tielbörger *et al*., 2014). Annuals are ideal for studying population dynamics due to their discrete generations, allowing relatively easy collection of demographic data (Gremer, 2023). Winter annuals typically germinate after the first autumn rains (10–20 mm), with the rainy season lengthening from the arid climate (December–March) to the mesic‐Mediterranean (October–May).

Scattered perennial shrubs provide distinct microhabitats in the four sites. They cover 5% of the area in A to 70% in MM (Tielbörger *et al*., 2014). In the arid climate under shrub canopies, nutrient availability (Holzapfel *et al*., 2006) and soil moisture (Tielbörger *et al*., 2014) can be enhanced. In more mesic climates, soil moisture is lower beneath shrubs compared with open areas (Tielbörger *et al*., 2014).

### Sampling design

Each site had five replicated blocks (10 m × 25 m), spaced at least five meters apart, with careful consideration to avoid occasionally occurring large rocks and trees. Within each block, five pairs of permanent quadrats (20 cm × 20 cm) were randomly placed with one quadrat per pair placed under a shrub canopy and one in an open area *c*. 50 cm from the shrub edge. This design accounted for the spatial micro‐heterogeneity created by perennial shrubs (Holzapfel *et al*., 2006; Metz & Tielbörger, 2016). One community is defined as the average of the five quadrats of a specific microhabitat (under shrubs or in open areas) in a block in each site (*N* communities = 40 = 4 sites × 2 microhabitats × 5 blocks; Fig. S1).

### Long‐term measure of species biomass

The long‐term fluctuations of species biomass in each community over the 13 yr were calculated using the variable Standing biomass (SB_*i,j*_; g m^−2^) of species *i*, in the year *j*.

$${\mathrm{SB}}_{i,j}=\frac{{\text{count}}_{i,j}\;{\text{biomass}}_{i,2022}}{\sum_{i=1}^{\mathrm{nsp}}{\text{count}}_{i,j}\;{\text{biomass}}_{i,2022}}\;{\text{Total Biomass}}_{j}$$

where ${\text{count}}_{i,j}$ is the number of individuals of species *i* in each community in the year *j*; ${\text{biomass}}_{i,2022}$ is the average biomass of an individual of species *i* in the year 2022 (i.e. average aboveground biomass of five randomly selected individuals of species *i* in each microhabitat of each site); and nsp is the total number of species in the quadrat. Mass proportion of species *i* in the community during the year *j* was thus $\frac{{\text{count}}_{i,j}\;{\text{biomass}}_{i,2022}}{\sum_{i=1}^{\mathrm{nsp}}{\text{count}}_{i,j}\;{\text{biomass}}_{i,2022}}$.

Total Biomass_*j*_ is the community biomass during the year *j* estimated using nondestructive estimates of aerial cover and mean vegetation height in each quadrat, and a calibration based on measured dried aboveground biomass (g m^−2^) from similar nearby vegetation (Tielbörger *et al*., 2014; GLOWA Jordan River project Tielbörger *et al*., 2016).

For more details on the estimation of SB_*i,j*_, see Notes S1.

### Statistical analyses

All species occurring at least once across the 13 yr were considered in the analyses (Table S1).

#### Community stability

We used the coefficient of variation CV to describe the natural year‐to‐year fluctuations of communities and how variable they are during a period without any major disturbance (Doak *et al*., 1998; de Bello *et al*., 2021). It is calculated as the ratio of the SD (*s*) to the mean ($\bar{X}$) (CV = $\frac{s}{\bar{X}}$). High CV values indicate high community variability, while lower values define stable communities. CV_c_ represented the temporal community variability calculated using Standing biomass $\sum_{i=1}^{\mathrm{nsp}}\sum_{j=2002}^{2015}{\mathrm{SB}}_{i,j}$. The straightforward interpretation of the CV has made it a common measure of temporal stability, allowing our findings to be easily compared with those of other studies (Conti *et al*., 2023; Lisner *et al*., 2024).

#### Decomposition of community stability

We used the comstab R package (Segrestin *et al*., 2024) to decompose the community‐level temporal stability CV_c_ into the stability of an average species and the stabilizing effects of dominance Δ, asynchrony *ψ* and averaging *ω* (see Fig. 2; Notes S2 for definitions of the mechanisms) as follows:

$${\mathrm{CV}}_{c}={\mathrm{CV}}_{e}\;\Delta \;\psi \;\omega$$

CV_e_ represents the coefficient of variation (CV) for a theoretical species with mean abundance ($\bar{x}$_e_ $=\frac{1}{\mathrm{nsp}}$ $\sum_{i=1}^{\mathrm{nsp}}\bar{x}$_*i*_, where $\bar{x}$_*i*_ is the Standing Biomass of species *i* across all years). It is computed as CV_e_ = *a* ^1/2^ $\bar{x}$_e_^*b*/2–1^ where *a* and *b* are the parameters derived from the Taylor's power law (Taylor, 1961) between the species' mean abundances ($\bar{x}$_*i*_) to their variances (*s*_*i*_^2^) in the community as *s*_*i*_^2^ = *a* $\bar{x}$_*i*_^*b*^.

**Fig. 2:**
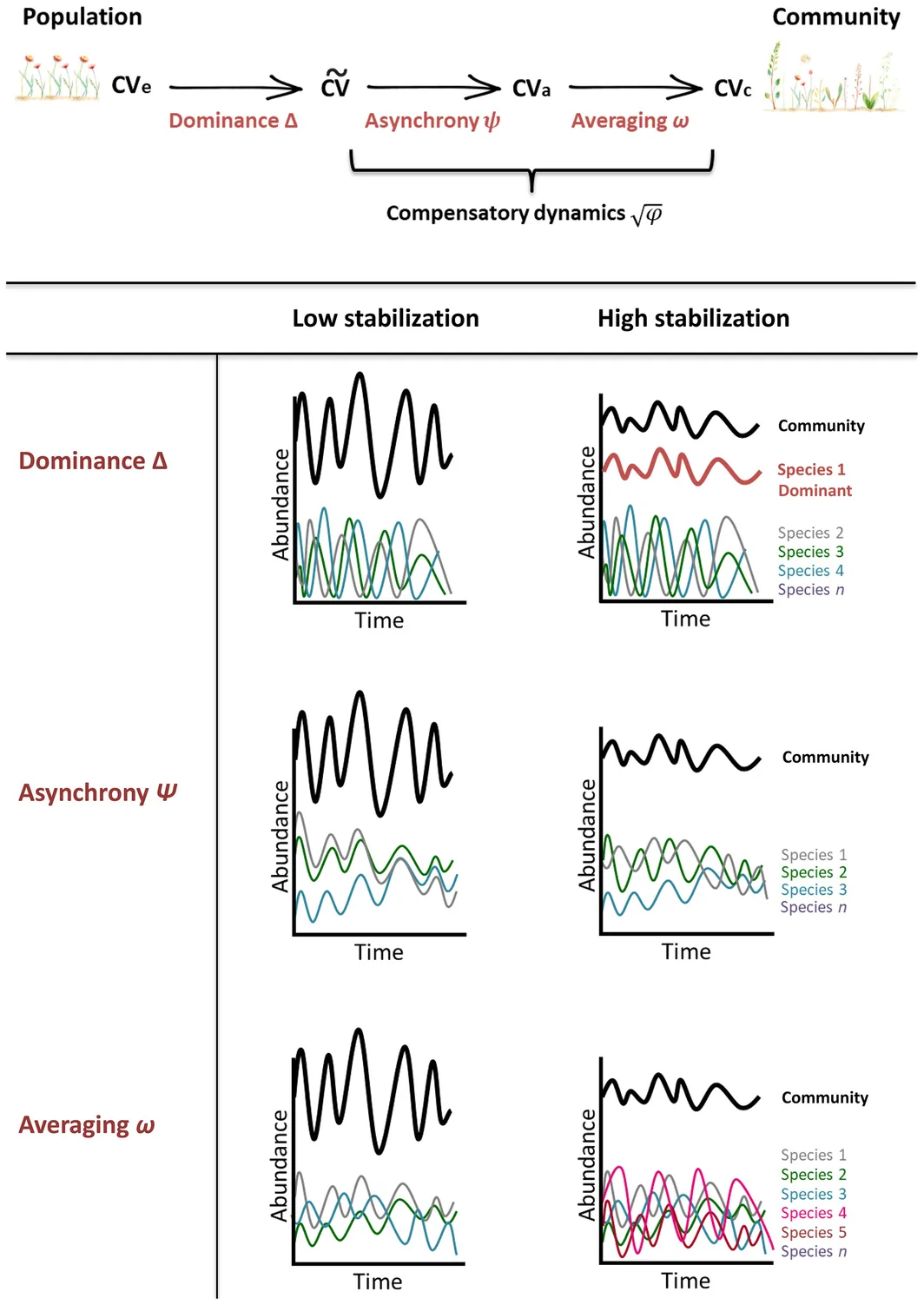
Conceptual framework for partitioning the drivers of community stability. Community variability (${\mathrm{CV}}_{c}$) is decomposed into the variability of a theoretical species of average abundance (${\mathrm{CV}}_{e}$, i.e. expected value of ${\mathrm{CV}}_{c}$ if all species had the same abundance and had perfectly synchronized temporal fluctuations) and three stabilizing components: the dominance effect (Δ), the asynchrony effect (*ψ*) and the averaging effect (*ω*). $\tilde{\mathrm{CV}}$ is the abundance‐weighted mean of species CV, and ${\mathrm{CV}}_{a}$ is the expected ${\mathrm{CV}}_{c}$ if the community was stabilized by species asynchrony only. Equations for Δ, *Ψ*, *φ*, and *ω* are provided in Supporting Information Notes S3. A, Arid; M, Mediterranean; MM, mesic‐Mediterranean; SA, semi‐arid.

Δ is calculated as the ratio of CV_e_ to the abundance‐weighted mean of species' coefficients of variation. It represents the dominance effect, capturing the stabilization provided by dominant species relative to the rest of the community. Δ < 1 means stabilization by dominants, and Δ > 1 mean destabilization by dominants, which happens when dominants are more variable than other species (*b* > 2 in Taylor's power law; Taylor, 1961).

The asynchrony index *ψ* measures the stabilization caused by asynchrony among species temporal fluctuations. Importantly, *ψ* is independent of community diversity. It is derived from a power transformation of the *φ* index (Loreau & de Mazancourt, 2008) and includes a scaling factor that constrains *ψ* to √1/2 (i.e. 0.71) when species fluctuate independently. If species fluctuate synchronously, *ψ* is higher than 0.71, and when species fluctuate antisynchronously, it is lower (Segrestin *et al*., 2024).

*ω* captures the averaging effect of species diversity, reflecting how community diversity contributes to stability given the observed level of species asynchrony. It is calculated as $\frac{\surd \phi }{\psi }$.

The overall stabilization of a community was calculated as $\frac{{\mathrm{CV}}_{c}}{{\mathrm{CV}}_{e}}$ (i.e. ∆ *ψ ω*). Stabilization values range from 0 (maximal stabilization) to 1 (minimal stabilization), while values exceeding 1 indicate destabilization of the community.

#### Effect of climate and microhabitat

To test whether community stability and its stabilizing mechanisms differ across climates, we used linear models (package lme4; Bates *et al*., 2015) with CV_c_, CV_e_, Δ, *ψ* and *ω* as response variables and site as predictor, for each microhabitat separately (i.e. in open areas and under perennial shrubs). Contrasts between sites and their confidence intervals were calculated with Tukey pairwise comparisons using the emmeans package (Lenth, 2022).

To test whether community stability and its stabilizing mechanisms differ between microhabitats, we used linear models (package lme4; Bates *et al*., 2015) with CV_c_, CV_e_, Δ, *ψ* and *ω* as response variables and microhabitat as predictor within each site (i.e. in A, SA, M and MM).

All the analyses and graphical outputs were performed using the R software v.4.2.2 (Wickham, 2016; R Core Team, 2022).

## Results

### Overall contribution of the ecological mechanisms to community stability

Overall stabilization from the population to the community level (calculated as $\frac{{\mathrm{CV}}_{c}}{{\mathrm{CV}}_{e}}$, that is ∆ *ψ ω,* but reported here as 1 − $\frac{{\mathrm{CV}}_{c}}{{\mathrm{CV}}_{e}}$ × 100% for easier interpretability; Fig. 3; Note: the steeper the slope, the higher the stabilization) increased from arid toward the more mesic climates and was higher in open microhabitats than under perennial shrubs: 43% in A, 62% in SA, 63% in M and 68% MM in the open microhabitats, and 38% in A, 49% in SA, 62% in M and 41% MM under shrubs.

**Fig. 3:**
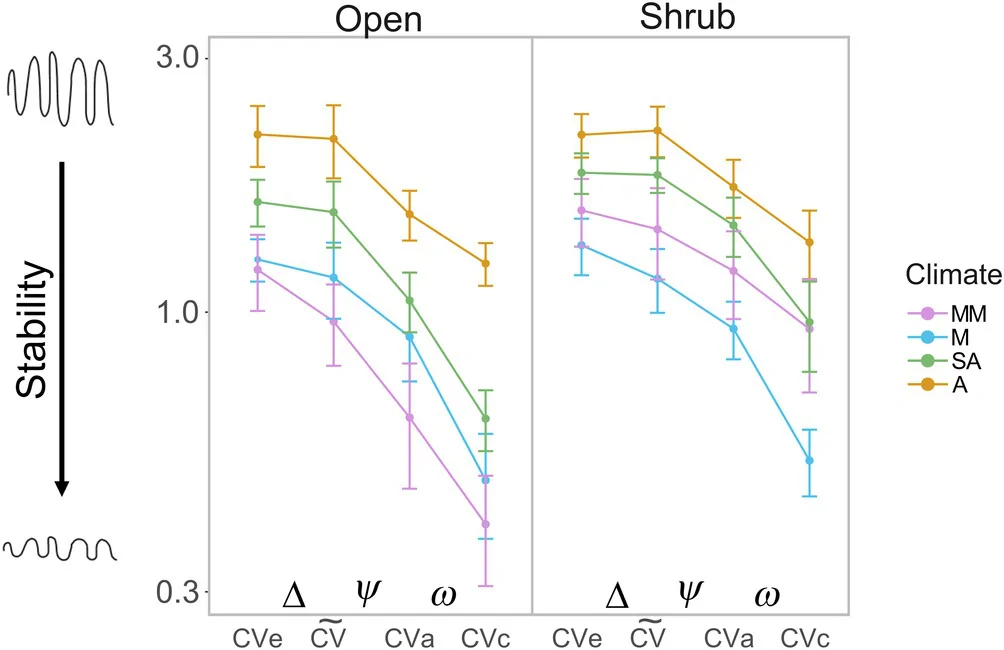
Effect of the aridity gradient on the different stability components of communities in open areas and under shrubs. The coefficient of variation of the average species (${\mathrm{CV}}_{e}$) is stabilized by the dominance (Δ), asynchrony (*ψ*) and averaging (*ω*) effects. $\tilde{\mathrm{CV}}$ is the abundance‐weighted mean of species' CV, and ${\mathrm{CV}}_{a}$ is the expected ${\mathrm{CV}}_{c}$ if the community was stabilized by species asynchrony only. The *y*‐axis is log transformed so the strength of each stabilizing mechanism is represented by the slope between two successive points: a steep negative slope means higher stabilization. Supporting Information Table S2 presents the numerical quantification of pairwise Tukey contrasts for the CVs, ∆, *ψ* and *ω*. A, Arid; M, Mediterranean; MM, mesic‐Mediterranean; SA, semi‐arid.

The overall contribution to the stabilization of plant communities (Figs 3, 4a–c; Note: the stabilization ranges from 0 being the highest stabilization to 1 being the lowest stabilization and > 1 denoting destabilization) was the weakest for the dominance effect (0.8–1.02), followed by stronger contributions of the asynchrony (0.6–0.8) and the averaging effects (0.5–0.8).

**Fig. 4:**
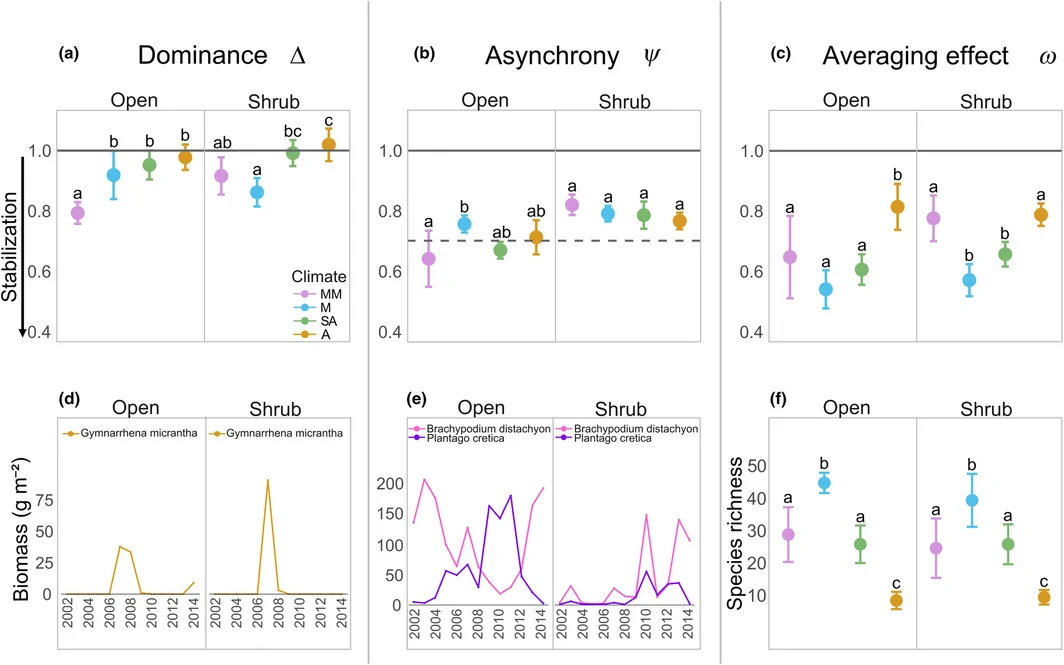
Stabilizing mechanisms across the aridity gradient. (a–c) Effect of the aridity gradient on the stabilizing mechanisms (a) dominance effect ∆; (b) asynchrony *ψ*; (c) averaging effect *ω* in open microhabitats and under shrubs. All indices range between 0 and 1 and the lower the value, the higher the stabilizing effect of each mechanism. The error bars represent 95% confidence intervals calculated from the linear model. Letters mark significance of pairwise Tukey contrasts between sites and correspond with numerical results in Supporting Information Table S2. (d) Temporal fluctuations in biomass over the 13 yr of the dominant species *Gymnarrhena micrantha* L. of a community in the arid site in open microhabitats and under shrubs. Most of the other years it remained dormant and showed outbreaks in some years in high abundance. (e) Temporal fluctuations in biomass over the 13 yr of the dominant species *Brachypodium distachyon* L. (colored in pink) and *Plantago cretica* L. (colored in purple) of a community in the mesic‐Mediterranean site in open microhabitats and under shrubs. (f) Mean species richness per block across all habitats along the aridity gradient. The error bars represent 95% confidence intervals calculated from the linear models conducted separately in each microhabitat (i.e. one model for in the open and one model for under shrub). Letters mark significance of pairwise Tukey contrasts between sites. A, Arid; M, Mediterranean; MM, mesic‐Mediterranean; SA, semi‐arid.

### Dominance Δ

Dominant species weakly stabilized the mesic communities in MM and M (Δ ≤ 1) and even tended to destabilize the arid communities in SA and A (Δ ≥ 1; Fig. 4a, and see positive slope for Δ in Fig. 3; Tables 1, S2). Overall, the stabilization by the dominance effect tended to be the same or slightly higher in the open areas than under shrubs, with a significant difference in MM (Figs 3, 4a; Table S3).

**Table 1:** Results of the overall linear model testing the effect of site (climates A, SA, M and MM) on the metrics ${\mathrm{CV}}_{c}$, ${\mathrm{CV}}_{e}$, $\tilde{\mathrm{CV}}$, ${\mathrm{CV}}_{a}$, Δ, *Ψ*, *ω* at two microhabitats (Open, Shrub).

	Open		Shrub	
	*F*‐value	*P*‐value	*F*‐value	*P*‐value
${\mathrm{CV}}_{c}$	**71.71**	**< 0.001**	**17.59**	**< 0.001**
${\mathrm{CV}}_{e}$	**24.46**	**< 0.001**	**17.38**	**< 0.001**
$\tilde{\mathrm{CV}}$	**23.22**	**< 0.001**	**22.93**	**< 0.001**
${\mathrm{CV}}_{a}$	**28.87**	**< 0.001**	**15.87**	**< 0.001**
Δ	**11.44**	**< 0.001**	**9.54**	**< 0.001**
*Ψ*	3.78	0.032	2.14	0.135
*ω*	**8.77**	**0.001**	**18.46**	**< 0.001**

Shown are *F*‐values and associated *P*‐values from separate ANOVAs for each response variable. Numerator (df_1_) and denominator (df_2_) degrees of freedom for all *F*‐tests are 3 and 16, respectively. Significant effects (*P* < 0.05) are highlighted in bold. A, Arid; M, Mediterranean; MM, mesic‐Mediterranean; SA, semi‐arid.

Dominant species were the most stable in each climate (scaling factor < 2, increasing from 1.67 in MM to 1.81 in A; Fig. S2). In MM, the dominant species *Brachypodium distachyon* L. and forb species *Plantago cretica* L. were significantly more stable than the rest of the species of the community (*t*‐test; *t*_38_ < −10.6; *P* < 0.001 for both species). In A, the dominant species *Gymnarrhena micrantha* L. had a larger variance than expected from the average trend and was therefore less stable than the other species of the communities (*t*_11_ = 4.2; *P* = 0.0016). It occurred rarely across the 13 yr and had outbreaks with high abundance in a few specific years (Fig. 4d).

### Asynchrony *ψ*

Asynchrony had a similar stabilizing effect on plant communities across all four climates (Figs 3, 4b; Tables 1, S2). However, the stabilizing effect of asynchrony was stronger in the open microhabitats than under the shrubs, with a significant difference in MM and SA and a marginally significant difference in M and A (Figs 3, 4b; Table S3).

Communities in open habitats of the MM, SA and A climates exhibited antisynchronous (negative) fluctuations (*ψ* ≤ 0.71; with *ψ* = 0.71 being the threshold separating positive and negative fluctuations), whereas all other communities showed partly synchronous (positive) fluctuations (*ψ* ≥ 0.71). In the MM open habitat, the dominant species *Brachypodium distachyon* L. and *Plantago cretica* L. fluctuated antisynchronously (Fig. 4e), resulting in higher overall asynchrony of the community (overall *ψ* < 0.71 for MM open communities; Fig. 4b). On the contrary, in the same MM climate under shrubs, these same dominant species fluctuated synchronously (Fig. 4e), resulting in lower overall asynchrony (*ψ* > 0.71 for MM communities under shrubs; Fig. 4b).

### Averaging effect *ω*

Averaging had a higher stabilizing effect on plant communities in the intermediate climates (SA, M) than in the extremes (A, MM; Figs 3, 4c; Tables 1, S2). This pattern followed the distribution of species richness being highest in the intermediate and lower in the extreme climates (Fig. 4c). Overall, the stabilization by the averaging effect did not differ between the microhabitats (Figs 3, 4c; Table S3).

### Population stability

The stability of an average species' population also decreased with aridity, with species being less stable (higher CV_e_) in the arid site compared with the other sites (Figs 3, 5a; Tables 1, S2). Under shrubs, species tended to be less stable (higher CV_e_) than in open areas, especially in the mesic‐Mediterranean climate (Figs 3, 5a; Table S3).

**Fig. 5:**
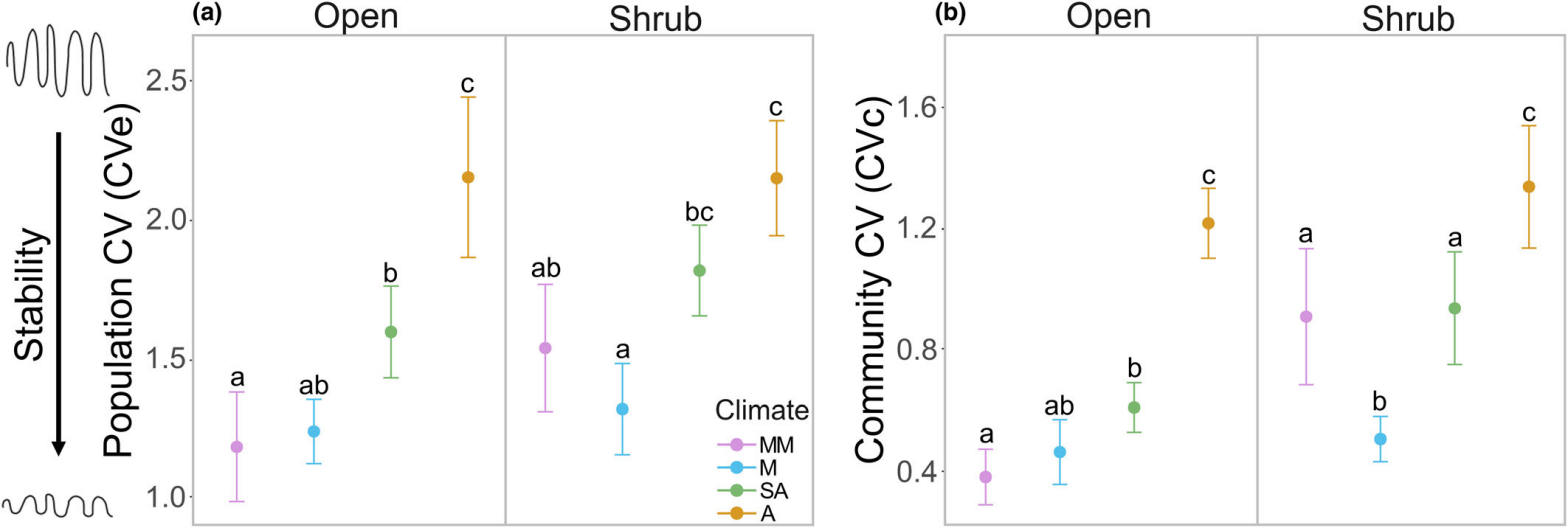
Population and community stability across the aridity gradient. Coefficient of variation of biomass at (a) population level (${\mathrm{CV}}_{e}$) and (b) community level (${\mathrm{CV}}_{c}$) as a response to the aridity gradient (i.e. across the sites MM, M, SA, A) in each microhabitat (i.e. in the open or under perennial shrubs). The error bars represent 95% confidence intervals calculated from the linear models conducted separately in each microhabitat (i.e. one model for in the open and one model for under shrub). Letters mark significance of pairwise Tukey contrasts between sites and correspond with numerical results in Supporting Information Table S2. A, Arid; M, Mediterranean; MM, mesic‐Mediterranean; SA, semi‐arid.

### Community stability

As a result of the three aforementioned mechanisms, stability of community biomass decreased toward arid climates, with communities being less stable (higher CV_c_) at the arid site compared with all other sites (Figs 1c, 3, 5b; Tables 1, S2). Under shrubs, communities tended to be less stable (higher CV_c_) than in open areas, especially in the semi‐arid and mesic‐Mediterranean climates (Figs 1c, 3, 5b; Table S3).

## Discussion

In the current debate on which of the three stabilizing mechanisms predominantly drive community stability, dominance effect, asynchrony in species fluctuations or averaging effect of species diversity (the latter two representing compensatory dynamics), our results support the importance of compensatory dynamics (Isbell *et al*., 2009; Loreau & de Mazancourt, 2013; Hautier *et al*., 2014; Blüthgen *et al*., 2016; Craven *et al*., 2018; Valencia *et al*., 2020; Zhao *et al*., 2022; Hou *et al*., 2023; Lisner *et al*., 2024). Most studies (Isbell *et al*., 2009; Loreau & de Mazancourt, 2013; Hou *et al*., 2023) show that the strength of compensatory dynamics increases with increasing species richness (Loreau & de Mazancourt, 2013; Ma *et al*., 2017). However, these studies were mostly using the *φ* index (Loreau & de Mazancourt, 2008) for calculating compensatory dynamics and, therefore, inevitably included the combined effects of species asynchrony and the averaging of species diversity (Thibaut & Connolly, 2013).

By disentangling the compensatory dynamics into distinct independent asynchrony and averaging effects, we showed that both are important stabilizing mechanisms of annual dryland communities, and that contrary to common assumptions, asynchrony in species fluctuations did not increase with increasing species richness, suggesting possible deviations from the expectations of the ‘Insurance hypothesis’ (Loreau, 2000). For instance, we identified the strongest asynchrony effect in the open mesic‐Mediterranean climate, which did not exhibit the highest species richness nor averaging effect. A meta‐analysis reported that species richness was unrelated to species asynchrony (Valencia *et al*., 2020). Likewise, recent studies decomposing compensatory dynamics (Zhao *et al*., 2022; Lisner *et al*., 2024) found no evidence that increasing species richness enhances negative temporal correlations among species. Our results emphasize the urgent need to disentangle asynchrony in species fluctuations and the averaging effect of species diversity to better understand the true relationship between biodiversity and stability.

Our findings suggest that the stabilizing effect of asynchrony might be a consistent ecological mechanism in annual plant communities in drylands, regardless of species richness or local climatic conditions. We found that antisynchrony in the open mesic‐Mediterranean climate highly stabilized the overall community. In this habitat, dominant species fluctuated antisynchronously, such as *Brachypodium distachyon* L. and *Plantago cretica* L. Negative temporal fluctuations among dominant species, have previously been reported (Bai *et al*., 2004). In more arid climates, asynchronous fluctuations (independent fluctuations or deviating from perfect synchrony) stabilized the communities as strongly as in the Mediterranean climates. This supports previous findings showing a consistent effect of species asynchrony across communities with varying species richness (Lisner *et al*., 2024). Our results suggest that in annual communities with lower species richness, stability can be preserved through asynchronous dynamics.

Our results also suggest that the role of biodiversity in maintaining stability might depend on the broader climatic context. Biodiversity has long been considered a general driver of community stability, with theoretical (Loreau & de Mazancourt, 2013; Wang *et al*., 2021) and empirical evidence (Craven *et al*., 2018; Hautier *et al*., 2018; Lepš *et al*., 2018; Yang *et al*., 2025) showing that greater species richness promotes stability. In our study, the averaging effect of species diversity was not consistently a strong driver of stability along the gradient and was the most pronounced in the intermediate climates of semi‐arid and Mediterranean, which supported the highest number of species. The high number of species increases the chances that fluctuations, even random, will compensate one another, thereby stabilizing the overall community, effect called ‘statistical inevitability of stability–diversity relationships’ by Doak *et al*. (1998), see also (Segrestin *et al*., 2024). This supports the idea that biodiversity–stability relationships are climate‐dependent (García‐Palacios *et al*., 2018). In the extreme open arid climate, asynchrony in species fluctuations was a more important driver of community stability than species diversity. This suggests that species‐poor communities are mostly stabilized by asynchrony in species fluctuations, where a few species compensate each other's biomass and stabilize overall community (Lisner *et al*., 2024). Under such high environmental stress, stability appears to depend more on differential species responses than on diversity *per se* (Loreau & de Mazancourt, 2013; Lepš *et al*., 2018). Moreover, under shrubs in the mesic‐Mediterranean, the averaging effect of species diversity was as weak as in the arid sites, despite higher species richness. This result illustrates the climate‐dependency of the effect of species diversity on stability of annual dryland communities.

Overall, our results are consistent with the global scaling relationship of Taylor's power law (Taylor, 1961; Gracia *et al*., 2026), which predicts that dominant species are the most stable. Nevertheless, although this relationship held even in the more arid and unpredictable climates, some dominant species still exhibited substantial variability, which could lead to destabilization of the overall community. This surprising result could potentially be attributed to dominants remaining viable through seed dormancy during unfavorable years (Tielbörger *et al*., 2012; ten Brink *et al*., 2020) and outbreaking in very high abundances during a favorable wet year, such as *Gymnarrhena micrantha* L. Dominant and nondominant species, can possess different traits and their life strategies can strongly differ (Arnillas *et al*., 2021; Padullés Cubino *et al*., 2022). This might imply that the same strategies adopted by the dominants to be well suited to the high environmental unpredictability could destabilize the overall community.

Overall, the decrease in community stability observed along the aridity gradient is in line with previous theoretical and empirical studies (Chesson & Huntly, 1989; Adler & Drake, 2008; Abrams *et al*., 2013; Gilbert *et al*., 2020; Ren *et al*., 2023; Wang *et al*., 2024). This reduced stability seems to stem primarily from lower average species' population stability in arid climates, where annual population sizes fluctuate strongly in response to extreme environmental fluctuations, such as brief favorable periods followed by prolonged dry spells (Noy‐Meir, 1973; Tielbörger *et al*., 2012; Gresse *et al*., 2026a). The overall degree of stabilization (i.e. the combination of dominance, asynchrony and species diversity effects) from population to the community level also decreased toward the arid climates, and therefore strengthened these differences among arid and mesic climates even more.

Our results further show that microhabitat conditions can influence the mechanisms stabilizing communities. Annual communities under perennial shrubs were overall less stable than in open areas, especially in the more mesic climates. This reduction in stability can be primarily explained by reduced species‐level stability under shrubs in mesic climates, likely caused by intensified competition for resources, such as light and water (Holzapfel *et al*., 2006; Tielbörger *et al*., 2014; Gresse *et al*., 2026a). On the contrary, in arid climates, shrubs can facilitate the conditions for annuals through increased nutrients and soil moisture (Liancourt *et al*., 2012). Accordingly, we did not observe reduced population stability under shrubs in arid systems, but rather similar stability to that found in open areas. Moreover, and interestingly, species were consistently more synchronous under shrubs than in open areas. Our findings imply that asynchrony is governed primarily by local conditions rather than climate (Gilbert *et al*., 2020). Species in open areas vs under shrubs may have different functional strategies (Bai *et al*., 2021; Gresse *et al*., 2026a), potentially resulting in distinct patterns of fluctuations. In all climates, under shrubs, rainfall variability might have been buffered, leading species to fluctuate more synchronously than in the open areas, ultimately contributing to lower overall community stability.

In conclusion, while our results support the importance of compensatory dynamics, they also stress the need to disentangle compensatory dynamics into the distinct independent effects of species asynchrony and diversity. We showed that, unlike the averaging effect of species diversity, asynchrony in species fluctuations did not diminish with decreasing species richness and consistently stabilized even species‐poor communities in arid, unpredictable climates, suggesting it as a potential general stabilizing mechanism of annual plant communities in drylands. Whether this pattern holds under other dryland climatic regimes remains to be tested. Identifying stabilizing mechanisms across gradients of aridity, climatic variability and unpredictability should help build more accurate predictions of community stability under future climate change.

## Competing interests

None declared.

## Author contributions

MM, JG, JL and JS contributed to the concept. KT and MS contributed to the data. JG, JS, LG, JL and PL contributed to the methods. JG contributed to the first draft. All authors contributed to writing the manuscript.

## Disclaimer

The New Phytologist Foundation remains neutral with regard to jurisdictional claims in maps and in any institutional affiliations.

## Supporting information


**Fig. S1** Schematic of the field sampling design.
**Fig. S2** Mean–variance relationship (Taylor's power law; Taylor, 1961) for all species in each habitat.
**Notes S1** Estimation of species biomass.
**Notes S2** Definitions of the ecological determinants of community stability.
**Notes S3** Equations of the ecological determinants of community stability.
**Table S1** List of species and families included in the study.
**Table S2** Results for pairwise comparisons of different levels of stability (coefficients of variation) and stabilizing mechanisms (*∆*, *ψ* and *ω*) between sites (fixed effect site) from Tukey *post hoc* test in each microhabitat.
**Table S3** Results of the overall model testing the effect of microhabitat (Open, Shrub) on the metrics ${\mathrm{CV}}_{c}$, ${\mathrm{CV}}_{e}$, ${\mathrm{CV}}_{a}$, $\tilde{\mathrm{CV}}$, Δ, *ω*, *Ψ* at each site.
Please note: Wiley is not responsible for the content or functionality of any Supporting Information supplied by the authors. Any queries (other than missing material) should be directed to the *New Phytologist* Central Office.

## Data Availability

Data are available from the Figshare Repository at doi: 10.6084/m9.figshare.31261375 (Gresse *et al*., 2026b).
