# Supplementary material for: Ecological determinants of stability in dryland plant communities
Source: New Phytol. 2026 Jul 29;252(5):1843–53. doi: 10.1111/nph.71453 (PMC13738672; doi:10.1111/nph.71453)
Supplement: Supplementary file 1 [file NPH-252-1843-s001.docx]

***New Phytologist* Supporting Information**
Article title: **Ecological determinants of stability in dryland plant communities**
Authors: Johanne Gresse^1,*^, Jules Segrestin^2^, Lars Goetzenberger^2,3^, Jan Leps^2,4^, Pierre Liancourt^5^, Merav Seifan^6^, Katja Tielboerger^1,7,8^, Maria Majekova^1,7,8^
Article acceptance date: 28 June 2026

**Table S1.** List of species and families included in the study. Species nomenclature follows <https://flora.org.il/en/plants/>

| **Site** | **Species** | **Family** |
| --- | --- | --- |
| A | *Carrichtera annua* | Brassicaceae |
| A | *Diplotaxis harra* | Brassicaceae |
| A | *Erucaria pinnata* | Brassicaceae |
| A | *Euphorbia chamaepeplus* | Euphorbiaceae |
| A | *Filago desertorum ** | Asteraceae |
| A | *Gymnarrhena micrantha* | Brassicaceae |
| A | *Hippocrepis unisiliquosa* | Fabaceae |
| A | *Picris longirostris ** | Asteraceae |
| A | *Plantago ovata* | Plantaginaceae |
| A | *Reichardia tingitana* | Asteraceae |
| A | *Schismus arabicus* | Poaceae |
| A | *Stipa capensis* | Poaceae |
| SA | *Adonis dentata* | Ranunculaceae |
| SA | *Aegilops peregrina* | Poaceae |
| SA | *Anagallis arvensis* | Primulaceae |
| SA | *Atractylis cancellata* | Asteraceae |
| SA | *Biscutella didyma* | Brassicaceae |
| SA | *Brachypodium distachyon* | Poaceae |
| SA | *Bromus fasciculatus* | Poaceae |
| SA | *Calendula arvensis ** | Asteraceae |
| SA | *Carrichtera annua* | Brassicaceae |
| SA | *Coronilla scorpioides* | Fabaceae |
| SA | *Crepis sancta ** | Asteraceae |
| SA | *Erodium malacoides* | Geraniaceae |
| SA | *Euphorbia chamaepeplus* | Euphorbiaceae |
| SA | *Filago contracta* | Asteraceae |
| SA | *Filago desertorum ** | Asteraceae |
| SA | *Hedypnois rhagadioloides ** | Asteraceae |
| SA | *Helianthemum salicifolium* | Cistaceae |
| SA | *Hippocrepis unisiliquosa* | Fabaceae |
| SA | *Hymenocarpos circinnatus* | Asteraceae |
| SA | *Linum strictum* | Linaceae |
| SA | *Medicago monspeliaca* | Fabaceae |
| SA | *Onobrychis crista-galli* | Fabaceae |
| SA | *Onobrychis squarrosa* | Fabaceae |
| SA | *Picris galilaea ** | Asteraceae |
| SA | *Reichardia tingitana* | Asteraceae |
| SA | *Rhagadiolus stellatus ** | Asteraceae |
| SA | *Rostraria cristata* | Poaceae |
| SA | *Stipa capensis* | Poaceae |
| SA | *Torilis tenella* | Apiaceae |
| SA | *Urospermum picroides ** | Asteraceae |
| SA | *Valantia hispida* | Caprifoliaceae |
| M | *Aegilops peregrina* | Poaceae |
| M | *Alyssum strigosum* | Brassicaceae |
| M | *Anagallis arvensis* | Primulaceae |
| M | *Avena sterilis* | Poaceae |
| M | *Biscutella didyma* | Brassicaceae |
| M | *Brachypodium distachyon* | Poaceae |
| M | *Bromus fasciculatus* | Poaceae |
| M | *Campanula hierosolymitana* | Campanulaceae |
| M | *Catapodium rigidum* | Poaceae |
| M | *Clypeola jonthlaspi* | Brassicaceae |
| M | *Convolvulus pentapetaloides* | Convolvulaceae |
| M | *Convolvulus siculus* | Convolvulaceae |
| M | *Coronilla scorpioides* | Fabaceae |
| M | *Crepis sancta ** | Asteraceae |
| M | *Crupina crupinastrum* | Asteraceae |
| M | *Erodium gruinum* | Geraniaceae |
| M | *Erodium malacoides* | Geraniaceae |
| M | *Euphorbia oxyodonta* | Euphorbiaceae |
| M | *Galium judaicum* | Rubiaceae |
| M | *Galium murale* | Rubiaceae |
| M | *Hedypnois rhagadioloides ** | Asteraceae |
| M | *Helianthemum salicifolium* | Cistaceae |
| M | *Hippocrepis unisiliquosa* | Fabaceae |
| M | *Hymenocarpos circinnatus* | Asteraceae |
| M | *Linum pubescens* | Linaceae |
| M | *Linum strictum* | Linaceae |
| M | *Lolium rigidum* | Poaceae |
| M | *Lomelosia palaestina* | Boraginaceae |
| M | *Lotus peregrinus* | Fabaceae |
| M | *Medicago monspeliaca* | Fabaceae |
| M | *Mercurialis annua* | Euphorbiaceae |
| M | *Onobrychis caput-galli* | Fabaceae |
| M | *Picris galilaea ** | Asteraceae |
| M | *Plantago afra* | Plantaginaceae |
| M | *Plantago cretica* | Plantaginaceae |
| M | *Pterocephalus plumosus* | Asteraceae |
| M | *Rhagadiolus stellatus ** | Asteraceae |
| M | *Scorpiurus muricatus* | Fabaceae |
| M | *Stachys neurocalycina* | Lamiaceae |
| M | *Theligonum cynocrambe* | Brassicaceae |
| M | *Thlaspi perfoliatum* | Brassicaceae |
| M | *Torilis tenella* | Apiaceae |
| M | *Trifolium campestre* | Fabaceae |
| M | *Trifolium dasyurum* | Fabaceae |
| M | *Trifolium pilulare* | Fabaceae |
| M | *Trifolium stellatum* | Fabaceae |
| M | *Urospermum picroides ** | Asteraceae |
| M | *Valantia hispida* | Caprifoliaceae |
| MM | *Aegilops peregrina* | Poaceae |
| MM | *Alyssum strigosum* | Brassicaceae |
| MM | *Anagallis arvensis* | Primulaceae |
| MM | *Atractylis cancellata* | Asteraceae |
| MM | *Avena sterilis* | Poaceae |
| MM | *Biscutella didyma* | Brassicaceae |
| MM | *Brachypodium distachyon* | Poaceae |
| MM | *Bromus fasciculatus* | Poaceae |
| MM | *Campanula hierosolymitana* | Campanulaceae |
| MM | *Catapodium rigidum* | Poaceae |
| MM | *Convolvulus pentapetaloides* | Convolvulaceae |
| MM | *Convolvulus siculus* | Convolvulaceae |
| MM | *Coronilla scorpioides* | Fabaceae |
| MM | *Crepis sancta* | Asteraceae |
| MM | *Crupina crupinastrum* | Asteraceae |
| MM | *Erodium gruinum* | Geraniaceae |
| MM | *Erodium malacoides* | Geraniaceae |
| MM | *Filago contracta* | Asteraceae |
| MM | *Galium judaicum* | Rubiaceae |
| MM | *Hedypnois rhagadioloides* | Asteraceae |
| MM | *Hippocrepis unisiliquosa* | Fabaceae |
| MM | *Linum pubescens* | Linaceae |
| MM | *Linum strictum* | Linaceae |
| MM | *Lolium rigidum* | Poaceae |
| MM | *Lomelosia palaestina* | Boraginaceae |
| MM | *Lotus peregrinus* | Fabaceae |
| MM | *Medicago monspeliaca* | Fabaceae |
| MM | *Mercurialis annua* | Euphorbiaceae |
| MM | *Picris galilaea* | Asteraceae |
| MM | *Plantago afra* | Plantaginaceae |
| MM | *Plantago cretica* | Plantaginaceae |
| MM | *Pterocephalus plumosus* | Asteraceae |
| MM | *Rhagadiolus stellatus* | Asteraceae |
| MM | *Scorpiurus muricatus* | Fabaceae |
| MM | *Senecio vernalis* | Asteraceae |
| MM | *Sherardia arvensis* | Rubiaceae |
| MM | *Theligonum cynocrambe* | Brassicaceae |
| MM | *Thlaspi perfoliatum* | Brassicaceae |
| MM | *Torilis tenella* | Apiaceae |
| MM | *Trifolium campestre* | Fabaceae |
| MM | *Trifolium dasyurum* | Fabaceae |
| MM | *Trifolium pilulare* | Fabaceae |
| MM | *Trifolium stellatum* | Fabaceae |
| MM | *Urospermum picroides* | Asteraceae |
| MM | *Valantia hispida* | Caprifoliaceae |
|  |  |  |

**Figure S1.** Schematic of the field sampling design. At each site, the sampling design consisted of five replicated blocks (10 m × 25 m), placed at least 5 m apart and arranged to avoid large rocks and trees. Within each block, five pairs of permanent quadrats were positioned, with one quadrat (20 cm × 20 cm) located beneath the canopy of a perennial shrub and the paired quadrat placed in the open area approximately 50 cm from the shrub edge. These communities were monitored for 13 years as part of the GLOWA Jordan River project Tielbörger et al. 2016; Tielbörger et al. 2014, providing annual species standing biomass data from 2002 to 2014.

**Notes S1.** Estimation of species biomass

$${SB}_{i,j}=\frac{{count}_{i,j}.{biomass}_{i,2022}}{\sum_{i-1}^{nsp} {count}_{i,j}.{biomass}_{i,2022}} . {Total Biomass}_{j}$$

The counts of individuals *count_i,j_* for each species *i* for each year *j* was done in each quadrat during the peak of the vegetative seasons (February-April) for the 2002-2015 period. Because the original SA and M sites used for these counts *count_i,j_* were later destroyed or became inaccessible, biomass in 2022 *biomass_i,2022_* was sampled in adjacent 50 m × 50 m slopes, located within 5 km, under similar soil and climate conditions. In 2022, the permanent quadrats used for *count_i,j_* were no longer available.

Total community biomass during the year *j* *Total Biomass_j_* was estimated across all species in each quadrat during the peak of the vegetative seasons (February-April) for the 2002-2015 period using aerial cover × mean vegetation height, and a calibration based on measured dried aboveground biomass (g/m^2^) from similar nearby vegetation (Tielbörger et al., 2014). Note that the calibration was missing for the years 2012, 2013 and 2014 in all sites, and for 2002 in SA, M and MM. Missing values were estimated in each site separately with the model: *Total Biomass_j_* = aerial cover × mean vegetation height × rainfall. A 5-fold cross-validation showed the model performed well from the arid to the mesic-Mediterranean site (R² = 0.4–0.9; RMSE = 16–198 g/m²). Note also that aerial cover and mean vegetation height were unavailable for 2004 at all sites, and for 2002, 2003, and 2005 at the arid site. Consequently, in these years at these sites, standing biomass *SB_i,j_* was simply computed as ${count}_{i,j}.{biomass}_{i,2022}$. Pearson correlations between ${count}_{i,j}.{biomass}_{i,2022}$ and *SB_i,j_* showed high correlation (r > 0.5 for 35 plots out of 40).

We repeated the same partitioning analysis excluding the years where *Total Biomass* was extrapolated. The results were qualitatively similar.

**Notes S2.** Definitions of the ecological determinants of community stability

***The stability of individual species' populations*** is a key factor from which community stability is derived, with communities comprised of stable populations being more likely to be stable (Lisner et al., 2024). When populations are less stable, the below-mentioned ecological mechanisms can compensate for this variability and maintain a high overall community stability (de Bello et al., 2021; Segrestin et al., 2024).

***The dominance effect***, in accordance with the mass-ratio hypothesis (Grime, 1998), represents the extent to which dominant species drive overall community stability (Hao et al., 2024; Hou et al., 2023; Sasaki & Lauenroth, 2011). In most of communities, according to Taylor’s power law (Taylor, 1961) which relates species’ temporal variances to a power function of their mean abundances and typically has a scaling coefficient below 2, the dominant species, i.e., those that account for the largest portion of biomass in a community, are more stable than the less abundant species. If the dominant species are stable, the total biomass of the community is also likely to be stable (Lisner et al., 2023).

***The asynchrony*** effect refers to the level of temporal compensations among species due to their asynchronous fluctuations (Blüthgen et al., 2016; Craven et al., 2018; Hector et al., 2010; Roscher et al., 2011; Valencia et al., 2020). Synchronous fluctuations of populations in time are usually linked to common species responses to year-to-year climatic variations (Loreau & de Mazancourt, 2013), where all species tend to increase their abundance in favorable years and decrease their abundance in unfavorable years. This leads to a highly variable year-to-year biomass production, which could in turn make the community vulnerable to an extreme event (e.g., several consecutive dry years). Any deviation from a perfect positive synchrony, coined asynchrony, therefore leads to a stabilization of a community (Craven et al., 2018; Lepš et al., 2018). Asynchrony comprises partially positive synchronous, independent, and anti-synchronous fluctuations (i.e., negative correlations in species’ fluctuations), with the latter leading to the strongest community stabilization (de Bello et al., 2021; Gonzalez & Loreau, 2009; Lepš et al., 2018). Asynchronous fluctuations in drylands can result from the storage effect operating through buffering population growth (Chesson, 2000; Chesson et al., 2004), for instance via dormancy, from species-specific responses to abiotic environmental conditions (Loreau & de Mazancourt, 2013), or from negative biotic interactions, particularly interspecific competition (Tilman, 1996).

***The averaging effect, also called the portfolio effect,*** quantifies the contribution of species diversity to temporal compensation among species. It is a pure statistical effect by which increasing the number of species increase the chance of slight differences in their temporal variation, resulting in an increased average stability of the community compared to having fewer species (de Bello et al., 2021; Doak et al., 1998; Isbell et al., 2009). This effect is measured at any given level of asynchrony (Segrestin et al., 2024). Even if species fluctuate independently and have equal abundances, increasing species diversity statistically leads to higher stabilization of total community biomass (de Bello et al., 2021; Isbell et al., 2009).

**Notes S3.** Equations of the ecological determinants of community stability

Δ ($\frac{\sum_{i=1}^{n} \text{CV}_{\text{i}}{\text{ }\text{p}}_{\text{i}}}{\text{CV}_{\text{e}}}$) is calculated by comparing CV_e_ with the mean of species CV weighted by their relative abundances where n is the number of species in the community and p_i_ is the relative abundance of species i. ψ is calculated as $\sqrt{\varphi}^{\alpha}$, where $\varphi=\frac{\sum_{i=1}^{n} \sum_{j=1}^{n} cov(x_{i}, x_{j})}{{(\sum_{i=1}^{n} \sigma_{x_{i}})}^{2}}$, with $x_{i}$and $x_{j}$the temporal biomasses of species *i* and *j*, and the double sum runs over all pairs of species in the community (Loreau & de Mazancourt, 2008). Compensatory dynamics $\sqrt{\varphi}$ are scaled by the exponent $\alpha$ to separate the effect of species asynchrony from the diversity-mediated averaging effect, following Segrestin et al. (2024), with $\alpha=\frac{log(\frac{1}{2})}{log(\sum_{i=1}^{n} {\sigma_{i}}^{2}/\sum_{i=1}^{n} {\sigma_{i})}^{2})}$. ω is calculated as $\frac{\sqrt{\varphi}}{\psi}$. The community-level fluctuations shown (black lines) are not meant to be realistic relative to the species-level dynamics; they are illustrative examples used solely to demonstrate how each of the three stabilizing mechanisms contribute to a shift from a relatively less stable to a relatively more stable community. These notes complement the Conceptual Diagram presented in the *Statistical Analyses* section of the Methods.

**Table S2.** Results for pairwise comparisons of different levels of stability (coefficients of variation) and stabilizing mechanisms (*∆*, *ψ*, and *ω*) between sites (fixed effect site) from Tukey post-hoc test in each microhabitat (in the open (a) and under shrubs (b)). ${CV}_{e}$ is mean coefficient of variation of individual species populations, $\tilde{CV}$ is mean of species *CV* weighted by their relative abundances, ${CV}_{a}$ is the expected community *CV* if the community was stabilized by species asynchrony only, and ${CV}_{c}$ is coefficient of variation at the community level. *∆* refers to dominance effect, *ψ* to asynchrony, and *ω* to averaging effect. The tests for stabilizing mechanisms compare individual slopes. The results correspond with Table 1 and Figure 3 (${CV}_{e}$, $\tilde{CV}$, ${CV}_{a}$, ${CV}_{c}$, *∆,* *ψ,* *ω*).

(a) In the microhabitat open:

|  | $\boldsymbol{CV}_{\boldsymbol{e}}$ | | $\tilde{\boldsymbol{CV}}$ | | $\boldsymbol{CV}_{\boldsymbol{a}}$ | | $\boldsymbol{CV}_{\boldsymbol{c}}$ | | ***Dominance ∆*** | | ***Asynchrony ψ*** | | ***Averag. eff. ω*** | |
| --- | --- | --- | --- | --- | --- | --- | --- | --- | --- | --- | --- | --- | --- | --- |
| Contrasts between sites | *t*-value | *P*-value | *t*-value | *P*-value | *t*-value | *P*-value | *t*-value | *P*-value | *t*-value | *P*-value | *t*-value | *P*-value | *t*-value | *P*-value |
| A - M | **7.17** | **<0.001** | **6.41** | **<0.001** | **6.39** | **<0.001** | **11.93** | **<0.001** | 1.72 | 0.344 | -1.2 | 0.633 | **4.91** | **<0.001** |
| A - MM | **7.6** | **<0.001** | **7.75** | **<0.001** | **9.05** | **<0.001** | **13.24** | **<0.001** | **5.4** | **<0.001** | 1.94 | 0.251 | **2.99** | **0.039** |
| A - SA | **4.36** | **0.002** | **3.87** | **0.007** | **4.84** | **<0.001** | **9.61** | **<0.001** | 0.75 | 0.874 | 1.17 | 0.653 | **3.74** | **0.009** |
| M - MM | 0.43 | 0.972 | 1.34 | 0.554 | 2.66 | 0.073 | 1.31 | 0.571 | **3.67** | **0.010** | **3.14** | **0.029** | -1.92 | 0.260 |
| M - SA | -2.81 | 0.055 | -2.54 | 0.092 | -1.55 | 0.434 | -2.32 | 0.135 | -0.97 | 0.767 | 2.38 | 0.123 | -1.17 | 0.651 |
| MM - SA | **-3.24** | **0.024** | **-3.87** | **0.007** | **-4.21** | **0.003** | **-3.63** | **0.011** | **-4.64** | **0.001** | -0.77 | 0.867 | 0.74 | 0.878 |

(b) In the microhabitat under shrubs:

|  | $\boldsymbol{CV}_{\boldsymbol{e}}$ | | $\tilde{\boldsymbol{CV}}$ | | $\boldsymbol{CV}_{\boldsymbol{a}}$ | | $\boldsymbol{CV}_{\boldsymbol{c}}$ | | ***Dominance ∆*** | | ***Asynchrony ψ*** | | ***Averag. eff. ω*** | |
| --- | --- | --- | --- | --- | --- | --- | --- | --- | --- | --- | --- | --- | --- | --- |
| Contrasts between sites | *t*-value | *P*-value | *t*-value | *P*-value | *t*-value | *P*-value | *t*-value | *P*-value | *t*-value | *P*-value | *t*-value | *P*-value | *t*-value | *P*-value |
| A - M | **6.82** | **<0.001** | **7.77** | **<0.001** | **6.55** | **<0.001** | **7.26** | **<0.001** | **4.8** | **0.001** | -1,13 | 0.678 | **6.38** | **<0.001** |
| A - MM | **5.01** | **<0.001** | **5.72** | **<0.001** | **4.36** | **0.002** | **3.74** | **0.009** | **3.14** | **0.029** | -2,5 | 0.098 | 0.35 | 0.985 |
| A - SA | 2.73 | 0.065 | **2.89** | **0.048** | 2.19 | 0.167 | **3.5** | **0.014** | 0.84 | 0.835 | -0,9 | 0.806 | **3.86** | **0.007** |
| M - MM | -1.81 | 0.307 | -2.05 | 0.211 | -2.19 | 0.169 | **-3.52** | **0.014** | -1.65 | 0.379 | -1,37 | 0.533 | **-6.03** | **<0.001** |
| M - SA | **-4.09** | **0.004** | **-4.89** | **<0.001** | **-4.36** | **0.002** | **-3.77** | **0.008** | **-3.96** | **0.006** | 0,23 | 0.996 | -2.52 | 0.094 |
| MM - SA | -2.29 | 0.143 | -2.84 | 0.053 | -2.17 | 0.174 | -0.24 | 0.995 | -2.3 | 0.139 | 1,6 | 0.405 | **3.51** | **0.014** |

**Table S3**. Results of the overall model testing the effect of microhabitat (Open, Shrub) on the metrics ${CV}_{c}$, ${CV}_{e}$, ${CV}_{a}$, $\tilde{CV}$, Δ, ω, Ψ at each site (climates A, SA, M, MM). Shown are F-values and associated p-values from separate ANOVAs for each response variable. Numerator (df₁) and denominator (df₂) degrees of freedom for all F-tests are 1 and 8, respectively. Significant effects (p < 0.05) are highlighted in bold.

| Variable | Site | | F-value | p-value | |
| --- | --- | --- | --- | --- | --- |
| ${CV}_{c}$c | | A | 1.34 | | 0.28 |
| ${CV}_{c}$c | | M | 0.54 | | 0.485 |
| $\boldsymbol{CV}_{\boldsymbol{c}}$**c** | | **MM** | **23.8** | | **0.001** |
| $\boldsymbol{CV}_{\boldsymbol{c}}$**c** | | **SA** | **12.97** | | **0.007** |
| ${CV}_{e}$e | | A | 0 | | 0.984 |
| ${CV}_{e}$e | | M | 0.81 | | 0.396 |
| $\boldsymbol{CV}_{\boldsymbol{e}}$**e** | | **MM** | **6.92** | | **0.03** |
| ${CV}_{e}$e | | SA | 4.54 | | 0.066 |
| ${CV}_{a}$a | | A | 2.51 | | 0.151 |
| ${CV}_{a}$C | | M | 0.14 | | 0.72 |
| $\boldsymbol{CV}_{\boldsymbol{a}}$**a** | | **MM** | **20.06** | | **0.002** |
| $\boldsymbol{CV}_{\boldsymbol{a}}$**a** | | **SA** | **15.46** | | **0.004** |
| $\tilde{\boldsymbol{CV}}$de | | A | 0.18 | | 0.681 |
| $\tilde{\boldsymbol{CV}}$C | | M | 0 | | 0.96 |
| $\tilde{\boldsymbol{CV}}$**de** | | **MM** | **10.43** | | **0.012** |
| $\tilde{\boldsymbol{CV}}$**e** | | **SA** | **5.49** | | **0.047** |
| ∆ | | A | 1.82 | | 0.214 |
| ∆ | | M | 1.91 | | 0.205 |
| **∆** | | **MM** | **14.79** | | **0.005** |
| ∆ | | SA | 1.87 | | 0.209 |
| ω | | A | 0.47 | | 0.511 |
| ω | | M | 0.68 | | 0.435 |
| ω | | MM | 3.4 | | 0.102 |
| ω | | SA | 3.06 | | 0.118 |
| ψ | | A | 3.72 | | 0.09 |
| ψ | | M | 4.03 | | 0.08 |
| **ψ** | | **MM** | **16.31** | | **0.004** |
| **ψ** | | **SA** | **24.12** | | **0.001** |

**Figure S2.** Mean-variance relationship (Taylor’s power law; Taylor 1961) for all species in each habitat. Each slope *b* (solid line) from the equation log(variance) = a + b × log(mean) is lower than 2. This means that the variance of the species increases more slowly with the mean than expected and that overall dominant species are more stable than rare species.

**References for Supporting Information**

Blüthgen, N., Simons, N. K., Jung, K., Prati, D., Renner, S. C., Boch, S., Fischer, M., Hölzel, N., Klaus, V. H., Kleinebecker, T., Tschapka, M., Weisser, W. W., & Gossner, M. M. Land use imperils plant and animal community stability through changes in asynchrony rather than diversity. *Nature Communications*, **7** (2016).

Chesson, P. Mechanisms of maintenance of species diversity. *Annual Review of Ecology, Evolution and Systematics*, **31**, 343–366 (2000).

Chesson, P., E Gebauer, R. L., Schwinning, S., Huntly, N., Wiegand, K., K Ernest, M. S., Sher, A., Novoplansky, A., & Weltzin, J. F. Resource Pulses, Species Interactions, and Diversity Maintenance in Arid and Semi-Arid Environments. *Weltzin Source: Oecologia*, **141**, 236–253 (2004).

Craven, D., Eisenhauer, N., Pearse, W. D., Hautier, Y., Isbell, F., Roscher, C., Bahn, M., Beierkuhnlein, C., Bönisch, G., Buchmann, N., Byun, C., Catford, J. A., Cerabolini, B. E. L., Cornelissen, J. H. C., Craine, J. M., De Luca, E., Ebeling, A., Griffin, J. N., Hector, A., … Manning, P. Multiple facets of biodiversity drive the diversity–stability relationship. *Nature Ecology and Evolution*, **2**, 1579–1587 (2018).

de Bello, F., Lavorel, S., Hallett, L. M., Valencia, E., Garnier, E., Roscher, C., Conti, L., Galland, T., Goberna, M., Májeková, M., Montesinos-Navarro, A., Pausas, J. G., Verdú, M., E-Vojtkó, A., Götzenberger, L., & Lepš, J. Functional trait effects on ecosystem stability: Assembling the jigsaw puzzle. *Trends in Ecology & Evolution,* **36**, 822–836 (2021).

Doak, D. F., Bigger, D., Harding, E. K., Marvier, M. A., O’Malley, R. E., & Thomson, D. The statistical inevitability of stability-diversity relationships in community ecology. *American Naturalist*, **151**, 264–276 (1998).

Gonzalez, A., & Loreau, M. The causes and consequences of compensatory dynamics in ecological communities. *Annual Review of Ecology, Evolution, and Systematics*, **40**, 393–414 (2009).

Grime, J. P. Benefits of plant diversity to ecosystems: Immediate, filter and founder effects. Journal of *Ecology,* **86**, 902–910 (1998).

Hao, J., Diao, H., Su, Y., Xu, P., Dong, K., & Wang, C. Short-term grazing reduced community stability by decreasing community-wide asynchrony and dominant species stability. *Ecosphere*, **15** (2024).

Hector, A., Hautier, Y., Saner, P., Wacker, L., Bagchi, R., Joshi, J., Scherer-Lorenzen, M., Spehn, E. M., Bazeley-White, E., Weilenmann, M., Caldeira, M. C., Dimitrakopoulos, P. G., Finn, J. A., Huss-Danell, K., Jumpponen, A., Mulder, C. P. H., Palmborg, C., Pereira, J. S., Siamantziouras, A. S. D., … Loreau, M. General stabilizing effects of plant diversity on grassland productivity through population asynchrony and overyielding. *Ecology*, **91**, 2213–2220 (2010).

Hou, G., Zong, N., & Shi, P. Shifts in dominant species modulate nitrogen effects on community temporal stability along a degradation gradient in Tibetan alpine grasslands. *Ecological Indicators*, **154** (2023).

Isbell, Polley, H. W., & Wilsey, B. J. Biodiversity, productivity and the temporal stability of productivity: Patterns and processes. *Ecology Letters*, **12**, 443–451 (2009).

Lepš, J., Májeková, M., Vítová, A., Doležal, J., & de Bello, F. Stabilizing effects in temporal fluctuations: management, traits, and species richness in high-diversity communities. *Ecology*, **99**, 360–371 (2018).

Lisner, A., Konečná, M., Blažek, P., & Lepš, J. Community biomass is driven by dominants and their characteristics – The insight from a field biodiversity experiment with realistic species loss scenario. *Journal of Ecology*, **111**, 240–250 (2023).

Lisner, A., Segrestin, J., Konečná, M., Blažek, P., Janíková, E., Applová, M., Švancárová, T., & Lepš, J. Why are plant communities stable? Disentangling the role of dominance, asynchrony and averaging effect following realistic species loss scenario. *Journal of Ecology,* **112**, 1832–1841 (2024).

Loreau, M., & de Mazancourt, C. Biodiversity and ecosystem stability: A synthesis of underlying mechanisms. *Ecology Letters*, **16**, 106–115 (2013).

Roscher, C., Weigelt, A., Proulx, R., Marquard, E., Schumacher, J., Weisser, W. W., & Schmid, B. Identifying population- and community-level mechanisms of diversity-stability relationships in experimental grasslands. *Journal of Ecology*, **99**, 1460–1469 (2011).

Sasaki, T., & Lauenroth, W. K. Dominant species, rather than diversity, regulates temporal stability of plant communities. *Oecologia*, **166**, 761–768 (2011).

Segrestin, J., Götzenberger, L., Valencia, E., de Bello, F. & Lepš, J. A unified framework for partitioning the drivers of stability of ecological communities. *Global Ecology and Biogeography*, **33**, e13828 (2024).

Taylor. L. R. Aggregation, variance and the mean. *Nature,* **189***,* 732–735 (1961).

Tilman, D. Biodiversity: Population Versus Ecosystem Stability. *Ecology,* **77**, 350-363 (1996).

Valencia, E., de Bello, F., Galland, T., Adler, P. B., Lepš, J., E-Vojtkó, A., van Klink, R., Carmona, C. P., rí Danihelka, J., Dengler, J., Eldridge, D. J., Estiarte, M., García-González, R., Garnier, E., Gómez-García, D., Harrison, S. P., Herben, T., Ibáñez, R., Jentsch, A., … Götzenberger, L. Synchrony matters more than species richness in plant community stability at a global scale. *Proceedings of the National Academy of Sciences of the United States of America,* **117**, 24345–24351 (2020).
